# Use of virtual reality to enhance rehabilitation experience in critically ill patients: a pilot feasibility study

**DOI:** 10.1186/s44158-026-00429-0

**Published:** 2026-07-14

**Authors:** Ian Buckley, Joanne Dowds, Cristiano Pargana, Seán McEntagart, Emily Naylor, Christopher Soraghan, Ignacio Martin-Loeches

**Affiliations:** 1 Physiotherapy Department, St James Hospital, Dublin, Republic of Ireland; 2 Surgery, Anaesthesia and Intensive Care Directorate, St James’s Hospital, Dublin, Republic of Ireland; 3 Department of Medical Physics and Bioengineering, St James Hospital, Dublin, Republic of Ireland; 4 Department of Intensive Care Medicine, Multidisciplinary Intensive Care Research Organization (MICRO), St James’ Hospital, St James’s Street, Dublin, D08 NHY1 Ireland; 5 School of Medicine, Trinity College Dublin, Dublin, D02 PN40 Ireland; 6 Trinity Centre for Biomedical Engineering, Dublin, Ireland

**Keywords:** Critical care, Virtual reality, Physiotherapy, Rehabilitation, Experience, Cycling

To the Editor,

Intensive care medicine has evolved from a primary focus on short-term survival, with greater attention being placed on longer-term health care outcomes [1]. Early intensive care unit (ICU) rehabilitation prevents functional decline, but patient engagement remains suboptimal. While in-bed cycling improves physical outcomes, integrating immersive virtual reality (VR) might enhance motivation. However, rigorous evidence regarding its ICU application remains limited. We report a pilot observational feasibility study evaluating the addition of immersive VR to standard ICU cycling.

Between May 2024 and June 2025, 20 VR-assisted cycling sessions were conducted among 19 adult ICU patients. Patients experiencing active delirium or lacking decision-making capacity were excluded. As an exploratory pilot, no formal sample size calculation was performed. Participants used a VR headset (Meta Quest 3) during routine seated cycle ergometry for up to 20 min (Fig. 1).

**Fig. 1:**
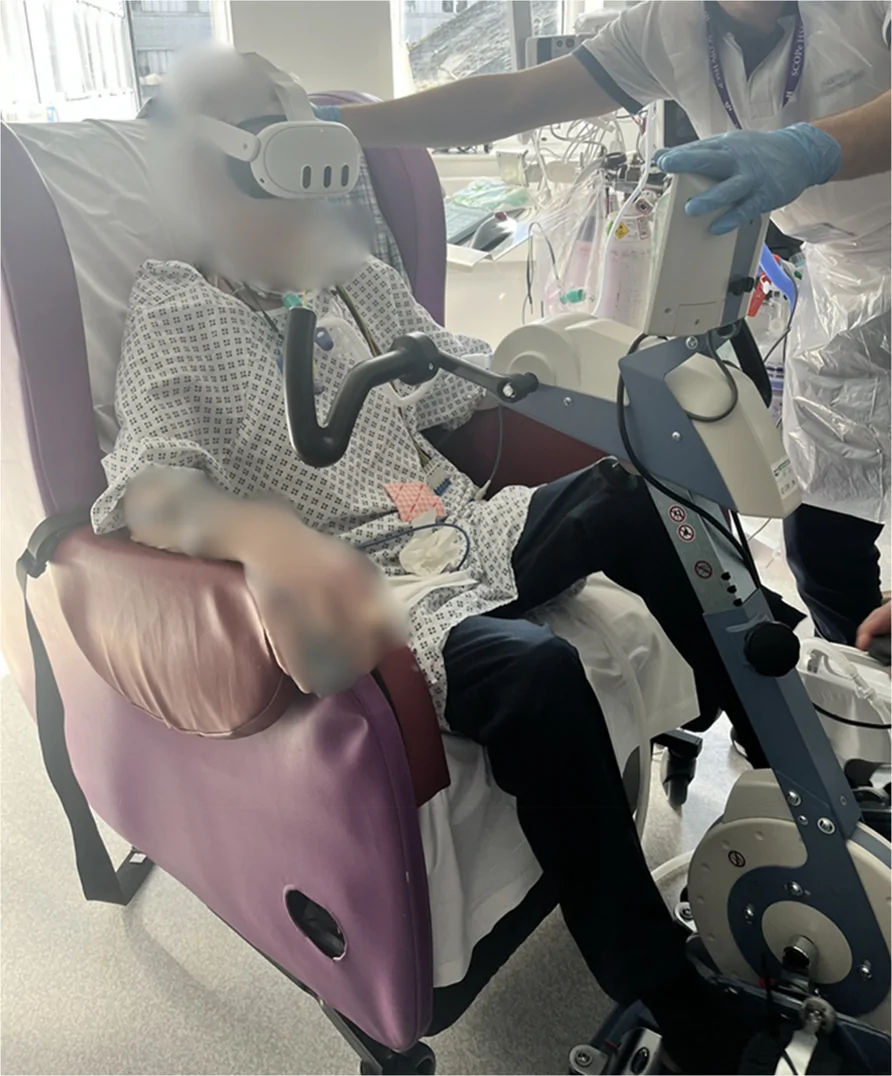
Use of virtual reality within an intensive care unit (permission obtained from the patient by a written form)

To address feasibility, the primary outcome was patient safety, defined as the absence of severe physiological deviations (> 20% baseline variation in heart rate or mean arterial pressure, SpO₂ < 85%) and no early discontinuation due to clinical instability. Secondary outcomes included patient satisfaction (Client Satisfaction Questionnaire-8 (CSQ-8)), usability (System Usability Scale (SUS)), and tolerability (Simulator Sickness Questionnaire (SSQ)). Data are reported as medians with interquartile ranges (IQR). Hemodynamic parameters remained stable throughout (Table 1), and no sessions required discontinuation due to physiological deterioration or safety breaches. Cycling durations ranged from 5 to 20 min (median 10); sessions concluding before the 20-min maximum were stopped due to anticipated physical fatigue rather than protocol-defined stopping criteria. Regarding tolerability, median SSQ scores were low (2, IQR 0–3) (Table 2). However, 3 of 20 sessions (15%) involved reports of mild general discomfort, alongside transient fatigue (*n* = 5) and increased salivation (*n* = 6). Median CSQ-8 scores (28.5, IQR 22–32) reflected high patient satisfaction, and system usability was rated “excellent” (SUS median 82.5, IQR 75–90).

**Table 1:** Physiological parameters before, during, and after VR-assisted cycling sessions (*n* = 20)

Physiological parameter	Pre-intervention median (IQR)	During intervention median (IQR)	Post-intervention median (IQR)
Mean arterial pressure (mmHg)	89 (77–96)	92 (81–100.5)	89 (80–97)
SpO₂ (%)	97 (96–99.5)	97 (96–98)	96 (96–98)
Heart rate (beats/min)	85 (78–91.5)	92 (84–103.5)	91 (86–96.5)
Respiratory rate (breaths/min)	18 (15–24)	25.5 (21.25–31.5)	21 (19.25–27.75)

**Table 2:** Patient-reported outcomes

Outcome measure	Median (IQR)	Interpretation
System Usability Scale (SUS)	82.5 (75–90)	Excellent usability
Client Satisfaction Questionnaire-8 (CSQ-8)	28.5 (22–32)	High patient satisfaction
Simulator Sickness Questionnaire (SSQ)	2 (0–3)	Minimal simulator-related symptoms

As per our findings, immersive VR-assisted cycling appears safe and yields high patient satisfaction. However, the 15% rate of mild general discomfort warrants cautious interpretation and rigorous monitoring in vulnerable critically ill cohorts. This study has significant methodological limitations. Without a sample size calculation, it is underpowered to detect clinical efficacy. Crucially, screening logs and demographic data were not prospectively maintained, precluding the assessment of recruitment rates, selection bias, or cohort characterization, which limits generalizability. Furthermore, broader constructs such as workflow feasibility were not systematically defined or measured. Additionally, while in-bed cycling provides known physiological benefits [2], rigorously evaluating intervention acceptability requires formal theoretical frameworks [3], and VR integration introduces novel clinical and technological complexities [4].

In conclusion, these preliminary findings suggest VR is a potentially safe, satisfying adjunct to ICU cycling. Given the limitations, these results are strictly hypothesis-generating. Future pre-registered, adequately powered trials with comprehensive screening logs, strict safety criteria, and robust primary endpoints are required.

## Data Availability

No datasets were generated or analysed during the current study.
